# Grade C molar-incisor pattern periodontitis subgingival microbial profile before and after treatment

**DOI:** 10.1080/20002297.2020.1814674

**Published:** 2020-09-13

**Authors:** Irina M. Velsko, Peter Harrison, Natalia Chalmers, Jennifer Barb, Hong Huang, Ikramuddin Aukhil, Luciana Shaddox

**Affiliations:** a Department of Archaeogenetics, Max Planck Institute for the Science of Human History, Jena, Germany; b Department of Periodontology, College of Dentistry, University of Florida, Gainesville, FL, USA; c Department of Periodontology, Trinity College, Dublin, Ireland; d NIH Clinical Center, Bethesda, MD, USA; e Clinical Center Nursing Department, Clinical Center, National Institutes of Health, Bethesda, MD, USA; f Center for Oral Health Research, University of Kentucky College of Dentistry, Lexington, KY, USA

**Keywords:** Aggressive periodontitis, subgingival microbiota, *A.a*., therapy, microarray

## Abstract

**Aim:** This study evaluated the influence of periodontal therapy on the microbiological profile of individuals with Grade C Molar-Incisor Pattern Periodontitis (C/MIP).

**Methods:** Fifty-three African-American participants between the ages of 5–25, diagnosed with C/MIP were included. Patients underwent full mouth mechanical debridement with systemic antibiotics (metronidazole 250 mg + amoxicillin 500 mg, tid, 7 days). Subgingival samples were collected from a diseased and a healthy site from each individual prior to treatment and at 3, 6, 12, 18 and 24 months after therapy from the same sites. Samples were subjected to a 16S rRNA gene based-microarray.

**Results:** Treatment was effective in reducing the main clinical parameters of disease. *Aggregatibacter actinomycetemcomitans* (*A.a*.) was the strongest species associated with diseased sites. Other species associated with diseased sites were *Treponema lecithinolyticum* and *Tannerella forsythia*. Species associated with healthy sites were *Rothia dentocariosa*/*mucilaginosa, Eubacterium yurii, Parvimonas micra, Veillonella* spp., *Selenomonas* spp., and *Streptococcus* spp. Overall, treatment was effective in strongly reducing *A.a*. and other key pathogens, as well as increasing health-associated species. These changes were maintained for at least 6 months.

**Conclusions:**Treatment reduced putative disease-associated species, particularly *A.a*., and shifted the microbial profile to more closely resemble a healthy-site profile. (Clinicaltrials.gov registration #NCT01330719).

## Introduction

Aggressive periodontitis (AgP) is a 1999 consensus term for a group of less frequently observed, often severe, rapidly progressive forms of periodontitis. It has previously been subdivided into localized and generalized forms [1], of which early age of onset, absence of systemic diseases, high rate of disease progression, and involvement of specific (localized) or multiple teeth (generalized) with a distinctive pattern of periodontal tissue loss are key diagnostic criteria [2]. Familial aggregation of disease and abnormalities of the host immune response are also frequently observed [1]. Localized forms of this disease (previously defined as localized aggressive periodontitis (LAP) tend to present in the circumpubertal period and exhibit a clinical presentation affecting first molar and incisor teeth. This disease has been recently re-classified into a Grade C periodontitis of molar and incisor pattern (C/MIP) [3].

Secondary features of Grade C periodontitis focus on the microbiology of the disease. A multitude of studies evaluated the microbiology of C/MIP; however, due to changing microbiological methods, disease terminology and diagnostic criteria over time, clinicians are faced with an evolving literature in which little definitive information has been determined. Nevertheless, strong associations have been reported between *Aggregatibacter actinomycetemcomitans (A.a.)* and localized forms of grade C periodontitis over many years [4–13]. Kaplan et al. [14] subdivided *A.a*. strains into three major phylogenetic lineages and noted that all three major lineages were recovered from C/MIP patients in some cases, suggesting diverse strains carry pathogenic potential. These associations may be complicated by the presence of various serotypes of *A.a*., which may be unequally pathogenic [15,16]. *A.a*. has also been detected in the subgingival microbiota of healthy children [5,11,16]. Based on PCR analysis of *A.a*. isolates and strains from periodontally healthy and diseased subjects [17], suggested that C/MIP was primarily associated with the highly leucotoxic clone of *A.a*., JP2, which was corroborated in a group of Moroccan subjects with C/MIP and GAP [7], and the JP2 clone is strongly associated with attachment loss [18,19].

It must be noted, however, that the presence of *A.a*., or the JP2 clone, in young individuals does not inevitably result in manifestation of C/MIP [20,21]. [22] reported 13 subjects who exhibited radiographic bone loss and detectable *A.a*. in their primary dentition and found progression of attachment loss in only three of the subjects after follow-up of 16 years. On the other hand, utilizing the Human Oral Microbe Identification Microarray (HOMIM) analysis of subgingival plaque samples from initially periodontally healthy adolescents and a follow-up period of 3 years, [23] identified that 16 of 63 subjects who were *A.a*. positive at baseline developed bone loss over the follow-up, in contrast to *none* of 71 subjects who were *A.a*. negative. Additional species were also elevated at diseased sites prior to bone loss and the authors concluded that detecting the presence of *A.a., Streptococcus parasanguinis* and *Fusobacterium alocis* together indicates sites of future bone loss in C/MIP.

In recent years, studies utilizing new technologies based on genomic analysis have revealed a more complex and diverse microbiota than aforethought and highlighted the potential for novel or not-yet-cultivable bacteria to change our understanding of the pathogenesis and treatment of grade C periodontitis [24]. For instance, a study characterizing the microbiota of 21 C/MIP subjects in Israel, noted that the subgingival flora of these individuals was mainly comprised of *Parvimonas micra, A.a.,*

*Fusobacterium nucleatum/Fusobacterium periodonticum* and *Tannerella forsythia* [25]. Our research group also previously characterized the microbiota from subgingival plaque samples taken from diseased and healthy sites in C/MIP subjects, as well as healthy siblings of C/MIP patients and healthy unrelated patients [11]. *A.a*. was shown to be the strongest species associated with C/MIP individuals, and site-specific to diseased sites in these subjects, as were *P. micra* and *Filifactor alocis*. In this study, other health-associated species were found to be associated with healthy sites/individuals, suggesting a possible protective role of healthy subgingival species in periodontal disease. Periodontal treatment was shown to substantially alter the species found at affected sites [26,27], and specific species including *A.a*. are reduced following treatment of C/MIP [13,28–30], but the long-term effects of treatment on the microbiology of C/MIP-affected and healthy sites in the same individuals are not yet known.

Our group has identified a cohort of African-American children diagnosed with C/MIP in northern Florida. Since this is a homogeneous group in terms of ethnicity, age, and patterns of disease, the objective of this study was to use 16S rRNA-based microarrays to run a comprehensive subgingival microbial characterization present in diseased individuals and the effects on periodontal therapy in these profiles.

## Methods

This study aimed to characterize the microbiota of a population of children, adolescents, and young adults with Grade C, molar-incisor-pattern (C/MIP) periodontitis before and after treatment. Children included in the study were recruited from the Health Departments of Leon County and Duval County in Tallahassee and Jacksonville, Florida, respectively, and from the University of Florida College of Dentistry patient pool from January 2007 to August 2011. The pool of patients used in this study is part of a large clinical trial (*Clinicaltrials.gov registration #NCT01330719)*. All children and their families were informed about the study protocol and signed an informed consent previously approved by the Institutional Review Board at University of Florida. Complete medical and dental histories were taken from all participants.

### Demographics of the study population

Subjects were included in the study if they were between 5 and 25 years old and were African-Americans with a diagnosis of C/MIP, characterized by the presence of at least two sites (incisor and/or first molar) and no more than two teeth other than first molar and incisors [1] with PD ≥ 5 mm in the presence of attachment loss and at least 2 mm bone loss detected on radiographic examination. Patients were excluded if they were smokers, had taken antibiotics within the preceding 3 months or any medications that could influence the characteristics of the disease (e.g. phenytoin, cyclosporine), had been diagnosed with any systemic diseases or conditions that could influence the progression and/or clinical characteristic of periodontal disease (e.g. diabetes or blood disorders), and/or were pregnant or lactating females. Exclusion criteria were applied at each visit.

## Clinical measurements

The following periodontal clinical parameters were taken by two calibrated examiners at the initial visit for all patients: pocket depth (PD), bleeding on probing (BoP); gingival margin position (GM); clinical attachment level (CAL); Plaque Index (PI) [31]: and presence or absence of visible plaque. Measurements were performed with a UNC-15 periodontal probe at six sites per tooth (mesio-buccal, buccal, distobuccal, mesio-lingual, lingual, and disto-lingual) and were recorded with computer software (Florida Probe®, Florida Probe Corporation, Gainesville, FL). Periapical and bite-wing x-rays were taken of the compromised teeth to confirm the C/MIP diagnosis. Examiners’ calibration was obtained when 80% of duplicate measures of probing depth and CAL were within 1 mm [32].

### Collection of bacterial subgingival biofilm

Bacterial subgingival biofilm was collected from one diseased site (DD, first molar or incisor with PD ≥ 5 mm, attachment loss and BoP) and from one healthy site (DH, non-affected molar or incisor; PD ≤ 3 mm, no BoP) from children with C/MIP. The area of collection was isolated with cotton rolls, and supragingival plaque was carefully removed. Subgingival samples were collected with a sterile endodontic paperpoint. Following sampling, the paperpoint was stored at −80^◦^C until processed.

### Periodontal therapy

Following examination, all patients received full mouth mechanical debridement, oral hygiene instructions, followed by a 7-day course of systemic antibiotics at baseline only (amoxicillin 500 mg (20 mg/kg/day) and metronidazole 250 mg (10 mg/kg), tid for 7 days – the dosage was adjusted as needed according to drug label instructions). Subjects were reevaluated at 3 and 6 months after baseline therapy and periodontal maintenance was performed (supra- and sub-gingival mechanical debridement and oral hygiene instructions) at all timepoints. New subgingival paperpoint samples were collected at 3 and 6 months after therapy from the same sites collected at baseline. Following sampling, the paperpoint was stored at −80^◦^C until processed.

### DNA isolation and microarray analysis

DNA was isolated from plaque samples with the use of a DNA Purification kit according to the manufacturer’s instructions (MasterPure, EPICENTRE Biotechnologies, Madison, WI). After purification, the DNA concentration was tested with the Nanodrop (ND-1000 Spectrophotometer, Nanodrop Technologies Inc., Wilmington, DE). A 200-ng quantity of each sample at a minimum concentration of 20 ng/μL was submitted to The Forsyth Institute for HOMIM (Human Oral Microbe Identification Microarray) analysis. [The method has been described in detail elsewhere [26]]. Briefly, 16S rRNA genes were PCR-amplified from DNA extracts with 16S rRNA universal forward and reverse primers. HOMIM uses 16S rRNA-based, reverse-capture oligonucleotide probes (typically 18 to 20 bases), which are printed on aldehyde-coated glass slides and probed with labelled PCR products (described above) which are hybridized in duplicate. A total of 369 independent probes were used to detect species/phylotypes. Several of the species names used for the probes have been changed or updated and are no longer used. Thus, we have kept the names as they were reported to us based on the HOMIM data set at the time the samples were run.

Microbial profiles were generated from image files of scanned arrays with a HOMIM online analysis tool (http://bioinformatics.forsyth.org/homim/). Detection of a particular taxon was determined by the presence of a fluorescent spot for that unique probe. A mean intensity for each taxon was calculated from hybridization spots of the same probe, and signals were normalized by comparison of individual signal intensities with the average of signals from universal probes and calculated as described previously [26]. Any original signal that was less than two times the background value was re-set to 1 and was assigned to the signal level 0. Signals greater than 1 were categorized into scores from 1 to 5, corresponding to ranked signal levels.

### Statistical analyses

Two HOMIM runs were conducted in this study, and in our analyses we included only common probes for both runs. Prevalence and abundance of individual species were analyzed between healthy and diseased sites at each timepoint and across timepoints by Wilcox test in R. Beta-diversity was calculated by the Bray–Curtis distance using the R package vegan v. 2.4–6 (https://CRAN.R-project.org/package=vegan), and subjected to principal coordinate analysis using the R package ape v. 2.5 (https://CRAN.R-project.org/package=ape). Significant differences in group clustering was tested with adonis (PERMANOVA) in the R package vegan. Shannon diversity was calculated with the package R package vegan v. 2.4–6. Significant differences between diseased (DD) and healthy (DH) sites as well as DD sites through time for number of species and Shannon diversity were calculated with Wilcox test in R. All plots were generated in R with ggplot2 v. 2.2.1 (https://ggplot2.tidyverse.org).

## Results

Fifty-three C/MIP patients were included: 42% males and 58% females, aged 6 to 19 (mean age 12.98 ± 3.99). Fifty-three diseased and 36 healthy sites were evaluated at baseline, 32 and 21 at 3 months, 28 and 12 at 6 months, and 30 and 4 at 12 months, respectively, and an additional 21 and 29 DD sites at 18 months and 24 months, respectively. Not all patients returned for an appointment at each timepoint, but all plaque samples collected at each timepoint were processed and analyzed. Reasons for missed appointments have been described previously; however, most were unknown, followed by forgetfulness, medical reasons, school activities or transportation issues [32]. Clinical parameters for all these sites at different timepoints are shown in Table 1. Differences were seen between DD and DH sites at baseline for all parameters (P < 0.0001), except for the presence of plaque. Significant reductions were observed after treatment in the DD sites, whereas reduction of PD, CAL and BOP were observed (P < 0.05), along with an overall reduction on the percentages of sites with PD > 4 mm and BOP up to 6 months (p < 0.01).

**Table 1.** Clinical parameters of diseased (DD) and healthy (DH) sites in grade C molar incisor pattern periodontitis (C/MIP) individuals before (baseline) and 3 and 6 months after treatment.

	Sites	Baseline	3 months	6 months	12 months	18 months	24 months
Mean PD (mm)	DD	5.82#	3.65***	4.18**	4.67***	3.35***	3.04***
	DH	2.21	2.67	2.8	2.8	2.43	2.53
Mean CAL (mm)	DD	4.04#	1.94**	2.53*	1.83***	0.64***	0.42***
	DH	0.036	0	0	0	0	0
% Sites with BOP	DD	100#	52.9***	17.6***	33.33***	10.8**	11.9***
	DH	0	0	0	0	0	0
% Sites with plaque	DD	57.80	41.20	35.30	16.67	29	28.4
	DH	39.30	16.70	31.70	0	0	0
%DB per patient	overall	8.10	3.90**	1.60***	2.34***	2.42***	2.23***

PD = pocket depth, CAL = clinical attachment level, BOP = bleeding on probing, DB = sites with pocket > 4 mm with concomitant bleeding on probing. ND = no data. *p < 0.05. **p < 0.01 and ***p < 0.0001 compared to baseline by Kruskal Wallis with Dunn’s multiple comparisons. #p < 0.001 compared to DH sites.

**Figure 1.**
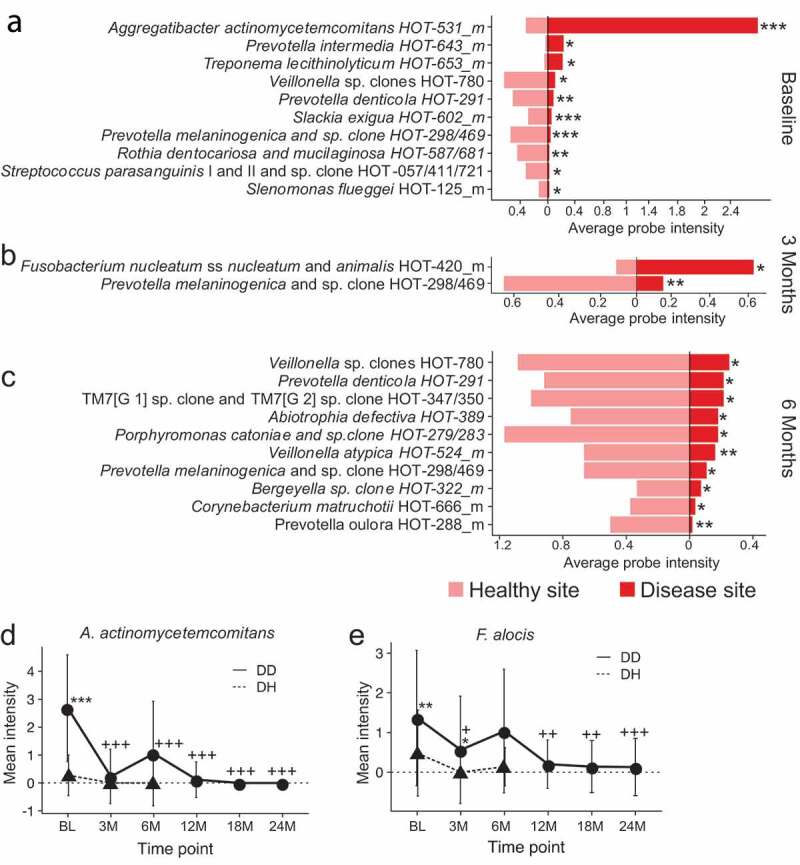
Differentially abundant species between disease and healthy sites before and after treatment in C/MIP patients. Species with a significant 2-fold difference in intensity between DD and DH sites at (a) Baseline, (b) 3 months post-treatment, and (c) 6 months post-treatment. (d) Mean intensity of *A.a*. in DD and DH sites through the study period. E. Mean intensity of *F. alocis* in DD and DH sites through the study period. DH site samples from 12 M, 18 M, and 24 M had too little material for HOMIM analysis. Values are mean ± SD. *P < 0.05, **P < 0.01, ***P < 0.001 between DD and DH. +P < 0.05, ++P < 0.01, +++P < 0.001 in DD compared to baseline. DD – diseased sites in C/MIP; DH – healthy sites in C/MIP.

Figure 1(a–c) shows the mean intensity of species with a statistically-significant absolute two-fold or more difference between diseased versus healthy sites for each time point in C/MIP participants. The highest fold-difference at baseline between the two sites is in the mean intensity of *A.a*. (P < 0.0001) (Figure 1(a)). Only two other species were found in 2-fold higher mean intensity at diseased sites, *Treponema lecithinolyticum* (P < 0.05) and *Prevotella intermedia* (P < 0.05). Species with higher mean intensity at healthy sites at baseline included *Veillonella* spp., *S. parasanguinis and Selenomonas flueggei*, (P < 0.05), *Prevotella denticola* and *Rothia dentocariosa/mucilaginosa* (P < 0.01), and *Prevotella melaninogenica* and *Slackia exigua* (P < 0.001) (Figure 1(a)).

After treatment, *A.a*. was infrequently detected in DD sites at 3 or 6 months follow-up, and at very reduced abundance. Three months after treatment, only *F. nucleatum* ss *nucleatum/animalis* was significantly enriched in disease sites (P < 0.05), while *P. melaninogenica* was enriched in healthy sites (P < 0.01) (Figure 1(b)). By six months after treatment, no species were significantly enriched in DD samples compared to DH sites, while 10 species were significantly enriched in DH sites (Figure 1(c)), including *Abiotrophia defectiva, Bergeyella* sp., *Corynebacterium matruchotii, Porphyromonas catoniae, P. denticola, P. melaninogenica*, the obligate epibiont TM7, and *Veillonella* sp. (P < 0.05), and *Prevotella oulora* and *Veillonella atypica* (P < 0.01).

Twenty-seven species had significantly different probe intensities between DD and DH sites at baseline, not considering 2-fold differences (Table 2). We found that 3 months after treatment, only four of these species were significantly different between DD and DH sites, with *F. alocis* and *Capnocytophaga spp*. higher in DD sites, and *Streptococcus anginosis/gordonii* and *Veillonella* spp. higher in DH sites. Six months post-treatment *Neisseria* cluster II had significantly higher probe intensity in DD sites, while six species had significantly higher intensity in DH sites. Numerous disease-associated species including *A.a*. and *F. alocis* had significantly lower probe intensity following treatment, while health-associated species including *P. denticola, P. oulora, S. anginosus/gordonii, S. parasanguinis*, and *R. dentocariosa*/*mucilaginosa* had higher probe intensity following treatment, although these species were not significantly more abundant at each time point.

**Table 2.** Average probe intensity of species significantly different between DD and DH sites at baseline.

	BL		3 M		6 M		12 M^†^	18 M^†^	24 M^†^
ProbeID	DD	DH	DD	DH	DD	DH	DD	DD	DD
*Campylobacter gracilis* HOT-623_m	2.698*	1.986	2.234	2.310	2.250	2.458	2.017+	1.571++	1.613++
*Aggregatibacter actinomycetemcomitans* HOT-531_m	2.679***	0.278	0.234+++	0.000	1.054+++	0.000	0.117+++	0+++	0+++
*Neisseria* Cluster II HOT-014/609/682/764	1.679*	1.028	1.688	1.619	1.607*	0.500	1.367	0.524++	0.387+++
*Tannerella forsythia* HOT-613	1.377**	0.500	0.406+	0.238	1.321	0.750	0.800	0.476++	0.613++
*Filifactor alocis* HOT-539_m	1.365**	0.481	0.563*+	0++	1.036	0.139	0.2++	0.143++	0.129+++
*Haemophilus* sp clone HOT-036_m	0.387***	0.139	0.156+	0.238	0.357	0.125	0.267+	0.143++	0.032+++
*Eubacterium*[11][G 5] *saphenum* HOT-759	0.34**	0.000	0.188	0.048	0.036	0.000	0.167	0.238	0+
*Prevotella intermedia* HOT-643_m	0.201*	0.028	NA	NA	0.071	0.000	0.033+	0+	0++
*Treponema lecithinolyticum* HOT-653_m	0.189*	0.042	0.141	0.024	0.143	0.000	0++	0.167	0.065
*Aggregatibacter segnis* and sp clone HOT-512/62_m	0.132+	0.306*	0.313+	0.619	0.071	0.250	0.067	0.095	0.097
*Streptococcus oralis* and sp clones HOT-064/707	1.754	2.388	2.968	3.761	2.928	3.167	3.033	3.190	3.484
*Parvimonas micra* HOT-111_m	1.518	2.069	1.781	1.928	2.196	1.625	1.850	2.167	2.177
*Capnocytophaga* sp HOT-335_m	0.755	1.444**	1.094*	0.286+++	0.500	0.5+	0.167++	0++	0.129++
*Eubacterium*[11][G 7] *yurii* HOT-377_m	0.745	1.986***	0.938	0.905++	0.518	1+	0.683	0.762	0.661
*Selenomonas infelix* and sp clones HOT-126/479/481/639_m	0.660	1.278*	0.781	1.190	0.625	1.083	0.383	0.214	0.032+++
*Streptococcus anginosus* and *gordonii* HOT-543/622_m	0.491	0.903*	1.109++	1.571*++	1.071+	1.083	1.133++	1.286++	1.581+++
*Selenomonas noxia* and sp clone HOT-130/140	0.358	0.722*	0.938+	1.000	0.250	0.917**	0.400	0.381	0.387
*Selenomonas* sp clones HOT-138/146_m	0.245	0.792**	0.438	0.881	0.125	0.583	0.200	0.095	0.129
*Streptococcus* sp strains HOT-070/071	0.226	0.583*	0.719+	1.143	0.571+	0.583	0.433	0.571	0.774
*Veillonella* sp clones HOT-780	0.094	0.556*	0.313	1.143*+	0.250	1.083*	0.067	0.238	0.419
*Prevotella denticola* HOT-291	0.075	0.444**	0.875+++	0.619	0.214	0.917*	0.267	0.143	0.452+
*Slackia exigua* HOT-602_m	0.047	0.25***	0.125	0.143	0.054	0.083	0.000	0.000	0.000
*Prevotella melaninogenica* and sp clone BE073 HOT-298/469	0.038	0.472***	0.125	0.619*	0.107	0.667*	0.067	0.048	0.097
*Rothia dentocariosa* and *mucilaginosa* HOT-587/681	0.019	0.389**	0.5+++	1.095+	0.357++	1.333*+	1+++	0.857+++	0.839+++
*Selenomonas flueggei* HOT-125_m	0.019	0.111*	0.156+	0.286	0.000	0.042	0.033	0.048	0.000
*Streptococcus parasanguis* I and II and sp clone HOT-057/411/721	0.019	0.278*	0.219+	0.524	0.357++	0.500	0.4+++	0.476++	0.387++
*Clostridiales*[F 2][G 1] sp clone HOT-075	0.000	0.111*	0.031	0.333	NA	NA	0.000	0.000	0.000
*Neisseria gonorrhoeae* and *polysaccharea* HOT-621/737	0.000	0.083*	0.063	0.143	0.107	0.167	0.000	0.000	0.000
*Prevotella oulora* HOT-288_m	0.000	0.153*	0.141++	0.476*+	0.018	0.5**+	0.000	0.000	0.129++

*P < 0.05, **P < 0.01, ***P < 0.001 between DD (diseased sites in C/MIP) and DH (healthy sites in C/MIP)

+P < 0.05, ++P < 0.01, +++P < 0.001, ++++P < 0.0001 compared to baseline within same group

^†^DH sites from 12 M, 18 M, and 24 M yielded too little material for HOMIM analysis

The mean intensity of highly DD site-specific species was further examined through time in both DD and DH sites to see how treatment affected the abundance of these species (Table 2). We found at DD sites that the abundance of *A.a*. was substantially reduced 3 months after treatment, and rebounded slightly at 6 months after treatment (Figure 1(d)). However, it then dropped to nearly undetectable levels and remained nearly undetectable for up to 24 months post-treatment. *F. alocis* showed a similar pattern, where the abundance dropped immediately following treatment, rebounded 6 months after treatment, and then dropped to nearly undetectable levels and remained so for up to 24 months post-treatment (Figure 1(d)). Both *A.a*. and *F. alocis* are much less abundant in DH sites than DD sites at all time points.

**Figure 2.**
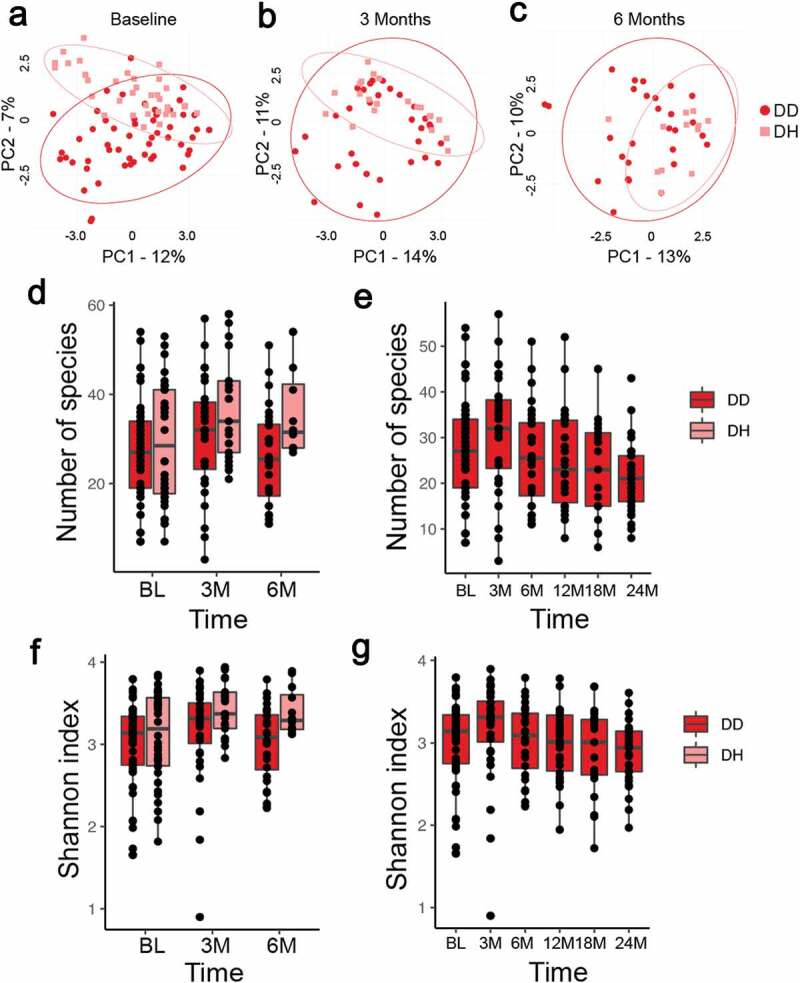
Bacterial diversity of DD and DH sites before and after treatment of C/MIP patients. (a) Principal coordinates analysis (PCoA) shows separation between clusters at baseline DD and DH sites is more profound than after treatment (P < 0.001). (b) PCoA of samples 3 months after treatment shows substantial overlap in bacterial profiles of DD and DH sites, but DH sites cluster more tightly (P < 0.05). (c) PCoA of samples 6 months after treatment shows substantial overlap in bacterial profiles of DD and DH sites, but DH sites cluster more tightly (P < 0.05). DD – red circles, DH – pink squares, and ellipses show 95% confidence intervals. (d) The number of species detected in DD sites at BL, 3, and 6 months after treatment is lower than in DH sites, but not significantly different. (e) The number of species detected in DD sites rises 3 months after treatment but falls at 6–24 months, but no differences are significant. (f) Shannon diversity in DD site samples at BL, 3 M, and 6 M is lower than DH sites, but not significantly different. (g) Shannon diversity increases in DD samples 3 months after treatment and then fall at 6–24 months after treatment, but no differences are significant. DD – diseased sites in C/MIP; DH – healthy sites in C/MIP.

We next examined the between-sample diversity (beta-diversity) of the plaque from disease and healthy sites at baseline, 3 months after treatment, and 6 months after treatment to see how community structure changed following treatment. Figure 2 shows Principal Coordinate Analysis (PCoA) at all three timepoints in DD (red circles) and DH (pink squares) sites. Note a greater separation of the DD vs DH ellipsis at baseline (P < 0.001) (Figure 2(a)) and then increasing overlap 3 months (Figure 2(b)) and 6 months (Figure 2(c)) after treatment. This suggests the disease-site bacterial profile shifts to a more healthy-like bacterial profile after treatment, and remains this way for at least 6 months. The DD site samples cluster less tightly than the DH site samples at each timepoint, suggesting that there is more variation in the bacterial profile of DD sites than DH sites, even after treatment.

We measured the within-sample diversity (alpha diversity) of DD and DH sites with both the number of observed species and the Shannon index, which incorporates species evenness. There was no significant difference in the number of species detected or Shannon index between DD and DH sites at BL, 3 months, or 6 months post-treatment, although the DH average was slightly higher at all times (Figure 2(d,f)). Diversity in DD sites initially increased at 3 months post-treatment, but then dropped to lower than baseline for both number of species detected and the Shannon index at all remaining timepoints (Figure 2(e,g)). There were no significant differences, but there was substantial variability in diversity between samples at all timepoints.

We further examined changes in the prevalence of species detected in DD and DH sites at baseline and following treatment. Twenty-seven species were significantly different between DD and DH sites at baseline (Table 3). Of these, eight were more prevalent in DD sites (*A.a., Campylobacter gracilis, Eubacterium saphenum, F. alocis, Haemophilus* spp., *P. intermedia, T. forsythia*, and *T. lecithinolyticum*), and the remaining 19 were more abundant in DH sites. We evaluated the prevalence of these 27 species in DD and DH sites at 3, 6, and 12 months post-treatment and found that most were no longer significantly different (Table 3). At 3 months, *F. alocis* was still significantly more prevalent in DD than DH sites (P < 0.05), but it reduced significantly from baseline after 6 months. In contrast, several species including *P. oulora, P. denticola, Prevotella melaninogenica*, and *Veillonella* spp. remained more prevalent in DH sites over time (P < 0.05).

**Table 3.** Prevalence of species significantly different between DD and DH sites at baseline.

	BL		3 M		6 M		12 M^†^	18 M^†^	24 M^†^
ProbeID	DD	DH	DD	DH	DD	DH	DD	DD	DD
*Aggregatibacter actinomycetemcomitans* HOT-531_m	81%***	17%	6.25%+++	0%	25%+++	0%	3%+++	0%+++	0%+++
*Campylobacter gracilis* HOT-623_m	92%*	78%	75%+	81%	75%+++	83%	67%++	48%+++	52%+++
*Neisseria* Cluster II HOT-014/609/682/764	64%	58%	62%	62%	61%	17%	50%	24%	16%
*Tannerella forsythia* HOT-613	52%*	31%	21%+	14%	61%	42%	33%	14%++	19%++
*Filifactor alocis* HOT-539_m	47%*	22%	18%*++	0%+	39%	8%	10%+++	5%+++	3%+++
*Haemophilus* sp clone HOT-036_m	43%***	8%	13%++	14%	32%	17%	17%+	10%++	3%+++
*Selenomonas infelix* and sp clones HOT-126/479/481/639_m	43%	61%	43%	52%	39%	58%	29%	24%	3%
*Prevotella intermedia* HOT-643_m	20%*	6%	0%+	0%	11%	0%	3%+	0%+	0%++
*Treponema lecithinolyticum* HOT-653_m	20%*	6%	13%	5%	21%	0%	0%++	10%	6%
*Eubacterium*[11][G 5] *saphenum* HOT-759	18%**	0%	9%	5%	4%	0%	7%	10%	0%+
*Streptococcus oralis* and sp clones HOT-064/707	77%	94%*	88%	100%	89%	100%	97%+	90%	94%
*Parvimonas micra* HOT-111_m	60%	80%*	75%	76%	79%	58%	73%	67%	71%
*Streptococcus anginosus* and *gordonii* HOT-543/622_m	43%	69%*	78%+	95%+	68%+	83%	77%++	76%+	81%++
*Eubacterium*[11][G 7] *yurii* HOT-377_m	40%	80%***	53%	43%++	39%	42%+	47%	43%	39%
*Capnocytophaga* sp HOT-335_m	38%	75%***	44%	19%+	25%	42%+	10%++	0%++	6%++
*Selenomonas* sp clones HOT-138/146_m	23%	47%*	31%	38%	11%	33%	13%	5%	6%
*Selenomonas noxia* and sp clone HOT-130/140	19%	38%*	41%+	38%	14%	58%**	17%	14%	19%
*Aggregatibacter segnis* and sp clone HOT-512/62_m	11%	33%*	31%+	33%	7%	8%	7%	5%	6%
*Streptococcus* sp strains HOT-070/071	11%	30%*	34%+	57%	36%++	33%	27%	24%	26%
*Veillonella* sp clones HOT-780	8%	25%*	18%	52%*+	11%	41%*	3%	10%	19%
*Slackia exigua* HOT-602_m	6%	33%**	16%	14%	11%	17%	0%	0%	0%
*Prevotella denticola* HOT-291	4%	22%**	38%+	19%	7%	33%*	13%	5%	16%
*Prevotella melaninogenica* and sp clone BE073 HOT-298/469	2%	27%***	6%*	28%*	4%	25%*	3%	5%	3%
*Rothia dentocariosa* and *mucilaginosa* HOT-587/681	2%	19%**	28%+	48%+	21%++	50%+	40%+++	33%+++	32%+++
*Selenomonas flueggei* HOT-125_m	2%	16%*	16%+	24%	0%	8%	3%	5%	0%
*Streptococcus parasanguis* I and II and sp clone HOT-057/411/721	2%	13%*	13%+	29%	21%++	25%	27%+++	19%++	19%++
*Clostridiales*[F 2][G 1] sp clone HOT-075	0%	8%*	3%	14%	0%	0%	0%	0%	0%
*Neisseria gonorrhoeae* and *polysaccharea* HOT-621/737	0%	8%*	3%	10%	7%	8%	0%	0%	0%
*Prevotella oulora* HOT-288_m	0%	11%*	15%*+	42%*++	4%	41%**+	0%	0%	13%++

*P < 0.05, **P < 0.01, ***P < 0.001 between DD (diseased sites in C/MIP) and DH (healthy sites in C/MIP)

+P < 0.05, ++P < 0.01, +++P < 0.001 compared to baseline within same group

^†^DH sites from 12 M, 18 M, and 24 M yielded too little material for HOMIM analysis

**Figure 3.**
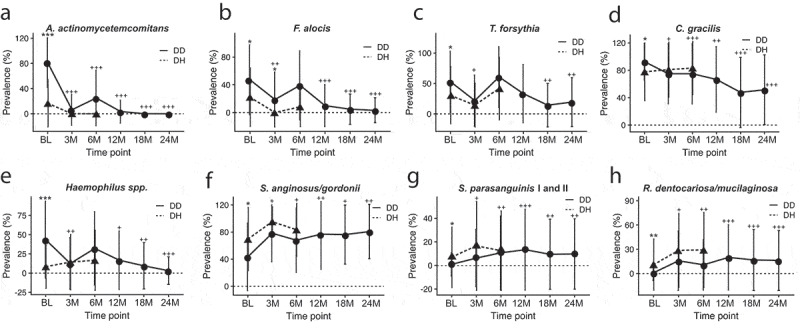
Changes in prevalence of health- and disease-associated species following treatment of C/MIP patients. The prevalence of disease-associated (a) *A.a*., (b) *F. alocis*, (c) *T. forsythia*, (d) *C. gracilis*, and E. *Haemophilus* spp. in DD sites drops following treatment, and remain less prevalent in DD sites than at baseline. In contrast, the prevalence of health-associated (f) *S. anginosus/gordonii*, (g) *S. parasanguinis*, and (h) *R. dentocariosa/mucilaginosa* rise following treatment and remain more abundant in DD sites than at baseline. DH site samples from 12 M, 18 M, and 24 M had too little material for HOMIM analysis. Values are mean ± SD. *P < 0.05, **P < 0.01, ***P < 0.001 between DD and DH. +P < 0.05, ++P < 0.01, +++P < 0.001 in DD compared to baseline. DD – diseased sites in C/MIP; DH – healthy sites in C/MIP.

When examining changes in prevalence relative to baseline in DD and DH sites of the same 27 species following treatment, we found a decrease in disease-associated species at DD sites from baseline, and an increase in prevalence of health-associated species (Table 3). For example, the prevalence of *A.a., F. alocis, T. forsythia, C. gracilis*, and *Haemophilus* spp. dropped significantly at 3 months post-treatment, rebounded slightly at 6 months, and then dropped and remained significantly lower than baseline at 12, 18, and 24 months post-treatment (Figure 3(a–e)). On the other hand, the prevalence of health-associated species *S. anginosus/gordonii, S. parasanguinis*, and *R. dentocariosa*/*mucilaginosa* increased significantly in DD sites following treatment and remained significantly higher through 24 months (Figure 3(f–h)). In contrast, species prevalence at DH sites was less variable after treatment (Table 3), with *Capnocytophaga* spp., and *Eubacterium yurii* dropping in prevalence from baseline to 6 months post-treatment (P < 0.05) and *P. oulora* and *R. dentocariosa*/*mucilaginosa* rising in abundance from baseline to 6 months post-treatment (P < 0.05). The unexpected increase in prevalence of disease-associated species in DH samples at 6 months post-treatment compared to baseline is not significant.

We determined species clusters that are associated with DD sites, DH sites, or common to both at BL, 3 months, and 6 months post-treatment. Species that were present in at least 50% of the DD sites and < 50% of the DH sites were specific to DD, while those that were present in at least 50% of the DH sites and < 50% of the DD sites were specific to DH, and species present in at least 50% of both DD and DH sites were common to both (Table 4). Only four species were present in at least 50% of DD sites, and those included *A.a., T. forsythia, F. periodonticum*, and *Prevotella* Cluster IV at BL, two species at 3 months post-treatment, and four species at 6 months, but only *T. forsythia* overlapped in these species between BL and 6 M. In DH sites, five species make the cluster in at least 50% of these sites at BL, 13 species at 3 months post-treatment, and 16 at 6 months, with *Capnocytophaga* sp. and *Selenomonas infelix* the only two species that were in the cluster at all three timepoints. The cluster shared by both DD and DH sites included 12, 16, and 16 species at BL, 3, and 6 months post-treatment, respectively, with *Campylobacter concisus/rectus, C. gracilis, Campylobacter showae, Catonella morbi, Gemella haemolysans, Gemella morbillorum, Haemophilus parainfluenzae, Parvimonas micra, Streptococcus constellatus/intermedius*, and *Streptococcus oralis* in the cluster at all three time points.

**Table 4.** Species clusters in C/MIP disease and healthy sites before and after treatment.

Time point	DD (>50% prevalent species)*	DD∩DH (>50% prevalent species)^†^	DH (>50% prevalent species)^‡^
BL	*Aggregatibacter actinomycetemcomitans* HOT-531_m	*Campylobacter concisus* and *rectus* HOT-575/748_m	*Capnocytophaga sp HOT-335_m*
	*Fusobacterium periodontium* HOT-201	*Campylobacter gracilis* HOT-623_m	*Eubacterium*[11][G 3] *brachy* HOT-557_m
	*Prevotella* Cluster IV HOT-658 693/714/782	*Campylobacter showae* HOT-763_m	*Eubacterium[*11][G 7] *yurii* HOT-377_m
	*Tannerella forsythia* HOT-613	*Catonella morbi* and sp clone HOT-164/165	*Selenomonas infelix* and sp clones HOT-126/479/481/639_m
		*Fusobacterium nucleatum ss nucleatum* and *animalis* HOT-420_m	*Streptococcus anginosus* and *gordonii* HOT-543/622_m
		*Gemella haemolysans* HOT-626_m	
		*Gemella morbillorum* HOT-046_m	
		*Haemophilus parainfluenzae* HOT-718	
		*Neisseria* Cluster II HOT-014/609/682/764	
		*Parvimonas micra* HOT-111_m	
		*Streptococcus constellatus* and *intermedius* HOT-576/644_m	
		*Streptococcus oralis* and sp clones HOT-064/707	
3M	*Eubacterium*[11][G 7] *yurii* HOT-377_m	*Campylobacter concisus* and rectus HOT-575/748_m	*Capnocytophaga granulosa* HOT-325_m
	*Prevotella* Cluster IV HOT-658 693/714/782	*Campylobacter gracilis* HOT-623_m	*Eikenella corrodens* and *Kingella denitrificans* and sp clone HOT-012/577/582_m
		*Campylobacter showae* HOT-763_m	*Fusobacterium periodontium* HOT-201
		*Catonella morbi* and sp clone HOT-164/165	*Kingella oralis* HOT-706_m
		*Fusobacterium* Cluster	*Neisseria elongata* HOT-598
		*Gemella haemolysans* HOT-626_m	*Prevotella oris* HOT-311_m
		*Gemella morbillorum* HOT-046_m	*Selenomonas infelix* and sp clones HOT-126/479/481/639_m
		*Granulicatella adiacens* HOT-534_m	*Streptococcus australis* HOT-073_m
		*Granulicatella elegans* HOT-596_m	*Streptococcus cristatus* and sp clone HOT-058/578
		*Haemophilus parainfluenzae* HOT-718	*Streptococcus salivarius* HOT-755_m
		*Neisseria* Cluster II HOT-014/609/682/764	*Streptococcus* sp strains HOT-070/071
		*Parvimonas micra* HOT-111_m	*Veillonella dispar* HOT-160*/Veillonella parvula* HOT-161
		*Streptococcus anginosus* and *gordonii* HOT-543/622_m	*Veillonella* sp clones HOT-780
		*Streptococcus constellatus* and *intermedius* HOT-576/644_m	
		*Streptococcus mitis* bv2 HOT-398_m	
		*Streptococcus oralis* and sp clones HOT-064/707	
6M	*Campylobacter* Cluster II HOT-580/748/763	*Campylobacter concisus* and *rectus* HOT-575/748_m	*Capnocytophaga granulosa* HOT-325_m
	*Neisseria* Cluster II HOT-014/609/682/764	*Campylobacter gracilis* HOT-623_m	*Cardiobacterium hominis* HOT-633_m
	*Streptococcus* Cluster III HOT-755/758/767/768	*Campylobacter showae* HOT-763_m	*Eikenella corrodens* and *Kingella denitrificans* and sp clone HOT-012/577/582_m
	*Tannerella forsythia* HOT-613	*Catonella morbi* and sp clone HOT-164/165	*Fusobacterium* Cluster
		*Eubacterium*[11][G 3] *brachy* HOT-557_m	*Fusobacterium periodontium* HOT-201
		*Fusobacterium nucleatum ss nucleatum* and *animalis* HOT-420_m	*Prevotella oris* HOT-311_m
		*Gemella haemolysans* HOT-626_m	*Propionibacterium propionicum* HOT-739_m
		*Gemella morbillorum* HOT-046_m	*Rothia dentocariosa* and *mucilaginosa* HOT-587/681
		*Granulicatella adiacens* HOT-534_m	*Selenomonas infelix* and sp clones HOT-126/479/481/639_m
		*Granulicatella elegans* HOT-596_m	*Selenomonas noxia* and sp clone HOT-130/140
		*Haemophilus parainfluenzae* HOT-718	*Solobacterium moorei* HOT-678_m
		*Parvimonas micra* HOT-111_m	*Streptococcus australis* HOT-073_m
		*Prevotella* Cluster IV HOT-658 693/714/782	*Streptococcus mitis* bv2 HOT-398_m
		*Streptococcus anginosus* and *gordonii* HOT-543/622_m	*Streptococcus salivarius* HOT-755_m
		*Streptococcus constellatus* and *intermedius* HOT-576/644_m	*Veillonella atypica* HOT-524_m
		*Streptococcus oralis* and sp clones HOT-064/707	*Veillonella dispar* HOT-160*/Veillonella parvula* HOT-161

*Species present in ≥ 50% of DD sites and < 50% of DH sites; † Species present in ≥ 50% of both DD and DH sites; ‡ Species present in ≥ 50% DH sites and < 50% DD sites. DD – C/MIP disease sites, DH- C/MIP healthy sites. Bold species are in the same group at all time points.

## Discussion

Here we show that periodontal therapy alters the bacterial community profile of localized aggressive periodontitis-affected sites towards a healthy-site profile, and that this change is sustained up to 24 months post-therapy. Certain C/MIP-associated species, particularly *A.a*. and *F. alocis*, that characterize disease sites, are substantially reduced in both abundance and prevalence following treatment, and do not rebound to pre-treatment levels, while health-associated species including *Streptococcus* spp., *Rothia* spp., and *Prevotella* spp. rise in prevalence and abundance after treatment. This suggests that one of the major benefits of periodontal therapy in C/MIP is a shift in the microbial profile to a less pathogenic, health-promoting community.

The high abundance and prevalence of *A.a*. in C/MIP disease sites agrees with numerous earlier reports that this species is strongly associated with C/MIP, especially in this study population [5,9,11,15]. PCR-based detection of *A.a*. in this cohort reported similar prevalence of *A.a*., and in particular the highly leukotoxic clone JP2 was dominant [20]. Non-surgical therapy reduced the prevalence JP2 and non-JP2 clones by specific PCR (Burgess, just like we detected with the microarray-based method here for *A.a*., indicating that HOMIM is highly concordant with traditional detection techniques [33]).

The rebound in *A.a*. prevalence and abundance at 6 months post-therapy is unexpected, but may be related to the ability of this species to invade epithelial cells and fibroblasts [34,35]. Re-emergence of *A.a*. from gingival tissues, where it would be protected from standard periodontal therapy, could explain the slight rebound in prevalence and abundance at 6 months. If the community were altered in a way that prevents *A.a*. from re-establishing in the biofilm, such as is suggested by the shift towards a healthy-site profile seen in our PCoA graphs, this may explain the subsequent drop of *A.a*. to nearly undetectable levels in C/MIP sites, after the 6 month timepoint. Alternatively, the reduced inflammation at DD sites following therapy also may prevent *A.a*. from accessing the nutrition it needs to sustain itself, and this may be why it drops again after the 6 month rebound. It is important to note that these patients received periodontal maintenance at all timepoints here, which included supra- and sub-gingival debridement with cavitron. This could also have helped the drop and the low prevalence of pathogenic species in the long term [36].

Other species associated with C/MIP were also reduced in prevalence and abundance following treatment, including *F. alocis* and *T. forsythia*, while health-associated species such as *R. dentocariosa*/*mucilaginosa, S. anginosus/gordonii* and *P. oulora* became much more prevalent. In the last several years, description of novel species in oral plaque samples from healthy and periodontitis-affected sites [37–39] have increased our awareness of the diversity of species present in plaque, and of the complicated interplay between microbial community and host. Although *A.a*. is clearly a dominant member of the community in this cohort, we showed that the overall community structure of C/MIP disease sites changes following treatment to more closely resemble that of a healthy site. Therefore, the role of other disease-associated species, including *F. alocis* and *T. forsythia*, should not be neglected. As technologies for detecting oral bacterial species improve, and as additional species are described, it will be important to reassess our understanding of the specific changes and their interplay occurring in disease-site plaque following periodontal therapy.

Following treatment, not only did disease-associated species become less prevalent and abundant, but health-associated species became more prevalent and abundant. This suggests that C/MIP treatment or prevention strategies should aim to promote health-associated species such as *S. sanguinis* [40], as well as to reduce disease-associated species. The healthier community at DD sites following treatment was able to sustain itself without re-emergence and re-establishment of disease-associated species such as *A.a., F. alocis*, and *T. forsythia* for up to 24 months following initial treatment and maintenance, which suggests a strong protective role of the health-associated community and the importance of a strict maintenance protocol. For instance, [40] showed that the absence of *S. sanguinis* was associated with grade C periodontitis. We also show here that *S. parasanguinis* I and II and *S. gordonii/anginosus* are less prevalent in diseased sites of C/MIP patients and do increase following treatment in these sites. Importantly, we see here that the microbial profile resembling a less pathogenic and increased health-associated species profile is in agreement with the clinical parameter reduction and maintenance of this disease seen here and reported long term in the larger cohort following treatment [32].

The differences in bacterial profile following treatment may be usable for site prognosis. When *A.a*. or other disease-associated species do not reduce in abundance, or quickly rebound, at a site, this could be an indicative of future disease progression. For instance, the specific consortium of *A.a., S. parasanguinis*, and *F. alocis* have been reported associated with development of bone loss in healthy individuals [23], although similar species-specific indicators of bone loss in treated sites has not yet been performed. The present study could not perform such an analysis here as the number of sites showing refractory disease or no *A.a*. reduction were very small and thus, provided insufficient power for such analysis. Follow-up studies examining whether specific species and their abundances can be used as diagnostic markers for disease progression following treatment are needed to determine the feasibility of using bacterial profiles as diagnostic or prognostic markers.

While HOMIM offers a broad overview of the species present in an oral microbial sample, it has several limitations that may be improved with more recent technologies such as HOMINGS [41]. The resolution for certain probes is not high enough to distinguish between closely-related species, such as *S. anginosis* and *S. gordonii*, or between certain species and closely related but unnamed species/strains, such as *S. oralis* and sp. clones HOT-064/707. This information will be important in the future, as there is substantial phenotypic variability within and between species and strains associated with oral health and disease [42–44]. Additionally, this method does not allow identification of novel or unexpected species, which may be important as discussed earlier.

A key factor in periodontitis progression is the microbial transcriptional profile, which we cannot account for with 16S rRNA-based microarray here. While transcriptional profiles of C/MIP site plaque have not yet been studied, those of progressing sites of chronic periodontitis show elevated expression profiles of *T. forsythia, P. intermedia*, and *C. gracilis* [45], species which were prevalent in our DD sites at baseline here but dropped following treatment. [46] reported slightly different results in grade C periodontitis sites. They found while the same bacterial genes are expressed in disease sites of different individuals, the species expressing those genes may differ between individuals. Therefore, the specific role of individual species and the interplay with other species in disease development and progression needs further investigation.

In summary, we demonstrated that initial mechanical periodontal therapy associated with systemic antibiotic and proper maintenance substantially alters the microbial profile of C/MIP sites to more closely resemble healthy sites, which is agreement with the clinical reduction of disease seen in the long term. While the mechanisms behind the transition and maintenance of a healthy microbial community following treatment of C/MIP sites are not yet known, specific species alterations provide a starting point for future investigations.
